# Regenerative capacity of human dental pulp stem cells versus their exosomes on surgically induced submandibular gland defects in rats

**DOI:** 10.1186/s12903-026-09337-9

**Published:** 2026-07-22

**Authors:** Basma A Hammad, Mohamed E Helal, Laila E Amin, Jilan Youssef, Rana El₋Qashty

**Affiliations:** 1 Oral Biology department, Faculty of Dentistry, Mansoura University, Mansoura, Egypt; 2 Oral biology department, Faculty of Dentistry, Horus University, New Damietta, Egypt; 3 Oral Medicine, Periodontology, Oral Diagnosis and Radiology department, Faculty of Dentistry, Mansoura University, Mansoura, Egypt

**Keywords:** Mesenchymal stem cells, Cell free therapy, Collagen-I, Aquaporin-5, Salivary gland tumors

## Abstract

**Background:**

Surgical excision of salivary gland (SG) tumors frequently results in glandular tissue loss and long-term functional impairment. Human dental pulp stem cells (hDPSCs) and their derived Exosomes (hDPSCs-Exos) have emerged as promising regenerative strategies. Therefore, the present study aimed to compare the regenerative capacity of hDPSCs versus their Exos on surgically induced defects in rats’ submandibular SGs.

**Methods:**

Seventy-two male Sprague Dawley rats were randomly assigned to four groups (n=18). Group I (negative control): no surgical intervention. The remaining rats underwent a circular defect preparation on the right submandibular SG, then the dissected areas were injected circumferentially with different treatments according to group assignment. Group II (positive control): 1ml phosphate buffered saline (PBS). Group III (hDPSCs group): 1×105 hDPSCs suspended in 1ml PBS, and group IV (hDPSCs-Exos): hDPSCs-Exos (20µg/ml) suspended in 1 ml PBS. Six rats from each group were euthanized at 3, 7, and 14 days postoperatively. Histological evaluation, immunohistochemical analysis (IHC) of Collagen-I (COL-I), and quantitative real time polymerase chain reaction (qRT-PCR) for aquaporin-5 (Aqua-5) gene expression were performed, followed by relevant statistical analyses.

**Results:**

The positive control group showed marked acinar disorganization and fibrosis. The hDPSCs-treated group demonstrated partial regeneration, whereas the exosome-treated group showed near-normal architecture by day 14. Statistical analysis showed significant differences between groups as COL-I expression decreased significantly over time, with the lowest levels in the exosome group indicating tissue regeneration without fibrosis. Aqua-5 expression increased significantly, showing superior molecular restoration in the exosome group approaching negative control baselines by day 14 that raises hope toward functional recovery followed by hDPSCs and finally positive control groups.

**Conclusions:**

Both hDPSCs and their exosomes promote regeneration of surgically injured submandibular glands. The exosomes-treated group demonstrated superior structural and molecular recovery approaching normal architecture, supporting their potentiality as a safer and effective cell-free regenerative therapy.

**Supplementary Information:**

The online version contains supplementary material available at 10.1186/s12903-026-09337-9.

## Background

Salivary gland (SG) malignancies, while uncommon, have great clinical significance due to their diverse histological characteristics and variable prognosis [1]. The submandibular glands are frequently affected by these cancerous outgrowths presenting as a painless mass, difficulties in swallowing, and compromised facial nerve function [2].

SGs malignancies range from low, intermediate to high grade that have the potential to recur and metastasize. Surgical excision is considered the proper treatment in case of low-grade ones, while a more aggressive treatment is needed for intermediate and high-grade ones including surgical excision combined with either chemotherapy, radiotherapy or both. All these treatments adversely affect the structure and function of SGs impacting patients’ quality of life which raises the need for an effective treatment that can regenerate the lost tissues restoring SGs structure and function [3].

Several experimental approaches have been investigated to regenerate SGs, including growth factor administration, gene therapy, tissue engineering, and stem cell–based therapies. Previous studies have demonstrated that stem cell therapy may improve glandular architecture, reduce fibrosis, and partially restore secretory function [4].

Mesenchymal stem cells (MSCs) are multipotent progenitor cells that can be harvested from various sources, including adipose tissue, amniotic membrane, cord blood and bone marrow. They possess the capability for adipogenic, osteogenic and chondrogenic differentiation [5]. Human dental pulp stem cells (hDPSCs), along with being multipotent, are ectomesenchymal cells originating from neural crest ones and have the potential to differentiate into a variety of cell types. Thus, hDPSCs have shown great potential to be used in regenerative medicine for treatment of various human diseases including dental related problems [6].

However, there are concerns associated with MSC-based therapy regarding immune rejection, and the potential to proliferate extensively which can lead to the formation of tumors. Moreover, MSCs promote tissue regeneration mainly through their paracrine signaling rather than direct cell differentiation [7].

Therefore, using cell free therapy instead of cell-based treatment could be a better option as a new therapeutic opportunity in regenerative medicine [8]. Stem cell derived secretome includes all the cellular products secreted by cells into their extracellular matrix (ECM) orchestrating various essential biological activities. Secretome is comprised of two main components: soluble factors and extracellular vesicles (EVs) [9].

The nano-sized extracellular vesicles, also termed exosomes (Exos), range in size between 30 nm and 150 nm and have therapeutic effects comparable to MSCs in various animal models, including myocardial ischemia-reperfusion injury, skin wounds, graft-versus-host disease, drug-induced liver injury, and bone and cartilage defects [10]. They contain growth factors, cytokines, deoxyribonucleic acid (DNA), ribonucleic acid (RNA), microRNAs (miRNAs), proteins, and lipids [11]. These exosomes possess the ability to engage in intercellular communication as they can enter cells and, modify the expression of genes in the recipient cells [12]. They play an essential role regulating immunity, and promoting blood vessels, and neural regeneration [13].

Collagen I (COL-I) is an essential component of the extracellular matrix in salivary glands, contributing structural support to the stromal tissue. It assists in identifying changes in the ECM, assessing the degree of fibrosis, and identifying mature COL-I apart from other types of collagens. This makes it useful for both experimental injury models and research on salivary gland diseases [14].

Aquaporin-5 (Aqua-5) is an essential water channel protein that is mostly found on the apical membranes of acinar and ductal cells in the salivary glands. It regulates the flow of water and saliva. Changes in the expression or location of Aqua-5 can make it harder for saliva to flow, which is a common sign of problems with the salivary glands.

This makes it a useful biomarker for diagnosing hyposalivation and evaluating glandular damage [15].

Rodents were selected for the present study because their SGs are anatomically well defined, functionally active, and histologically comparable to the human submandibular SGs, making them a widely accepted and reproducible experimental model for SGs research [16]. So, the aim of the present study was to evaluate the regenerative capacity of hDPSCs versus their Exos on surgically induced submandibular gland defects in rats.

## Methods

### Ethics approval

All experimental procedures were performed in compliance with the protocol approved by the Ethical Committee of Mansoura University Animal Care and Use Committee (MU-ACUC), Mansoura University, Mansoura, Egypt, with the ID: MU-ACUC (DENT.Phd.24.11.13). All biosecurity and biosafety protocols were executed in accordance with Alderman et al. [17] and following the Animal Research: Reporting of In Vivo Experiments (ARRIVE) guidelines.

### Animals and sample size calculation

Sample size calculation was determined based on the mean changes in histology and immunohistochemistry as an indicator for the regeneration of surgically induced defects in SGs obtained from a former research [18]. G*power version 3.1.9.4 was used to estimate sample size built on effect size of 0.5026, 2-tailed test, α error = 0.05 and power = 95.0%. The sample size calculation estimated a minimum of six animals per group.

Accordingly, seventy-two, healthy, male Sprague Dawley rats, weighing 250 to 300 g, were purchased from the Medical Experimental Research Center (MERC) at Mansoura University, Mansoura, Egypt, where all experimental procedures were performed. The rats were kept in individual cages (20 cm × 40 cm), six rats per cage, and had free access to water ad libitum and standard pelleted food. They were maintained in a 12-hour light/dark cycle, within a room at 26 °C and roughly 65–70% humidity.

### Study design

The rats were randomly assigned to four groups (*n* = 18). 

#### Group I (negative control)

Rats had no surgical intervention; they were housed in the same environmental conditions and euthanized following the same timeline as other groups. 

#### Group II (positive control)

A circular incision was created on the right submandibular SGs, then the inner margin of surgical site was injected circumferentially with 1 ml phosphate-buffered saline (PBS; Cat #10010023, Gibco, Thermo Scientific, Waltham, USA). 

#### Group III (hDPSCs-treated)

Surgical wounds were created as in group II and subsequently injected with hDPSCs (1 × 10^5^) suspended in 1 ml PBS [19]

#### Group IV (hDPSCs- Exos)

A surgical defect was created as in group II but injected with hDPSCs-Exos (20 µg/ml) suspended in 1 ml PBS [20].

Six rats from each group were euthanized at each of the three intervals (3, 7, and 14 days, postoperatively) by anesthesia overdose through intraperitoneal injection of sodium pentobarbital (120 mg/kg, alfa chemical group, Cairo, Egypt). Submandibular SGs were then immediately collected and prepared for histopathological and molecular analysis. The carcasses were then sealed in plastic bags and incinerated.

### Preparation of the rat SGs’ surgical defects [19]

Animals were anesthetized using xylazine intra-peritoneal injection (5–10 mg/kg; Cat#080905, Bimeda, California, USA) and ketamine hydrochloride (75 mg/kg; Cat # K2753, Sigma-Aldrich, Burlington, US). The surgical field was disinfected with betadine. A No. 15 surgical blade was then used to create a vertical incision in the submandibular region, followed by lifting the superficial fascia using toothed Adson Kocher tissue tweezer that was subsequently dissected with dissecting scissors to reveal the underlying gland.

A circular cut was performed by a tissue punch of dimensions 5 mm width × 3 mm depth, on the right glands. The incised tissue was grasped by a toothed tweezer and cut using dissecting scissors. The surgical region was rinsed with normal saline. The surgical area was injected circumferentially with different treatments according to the assigned group.

**Fig. 1:**
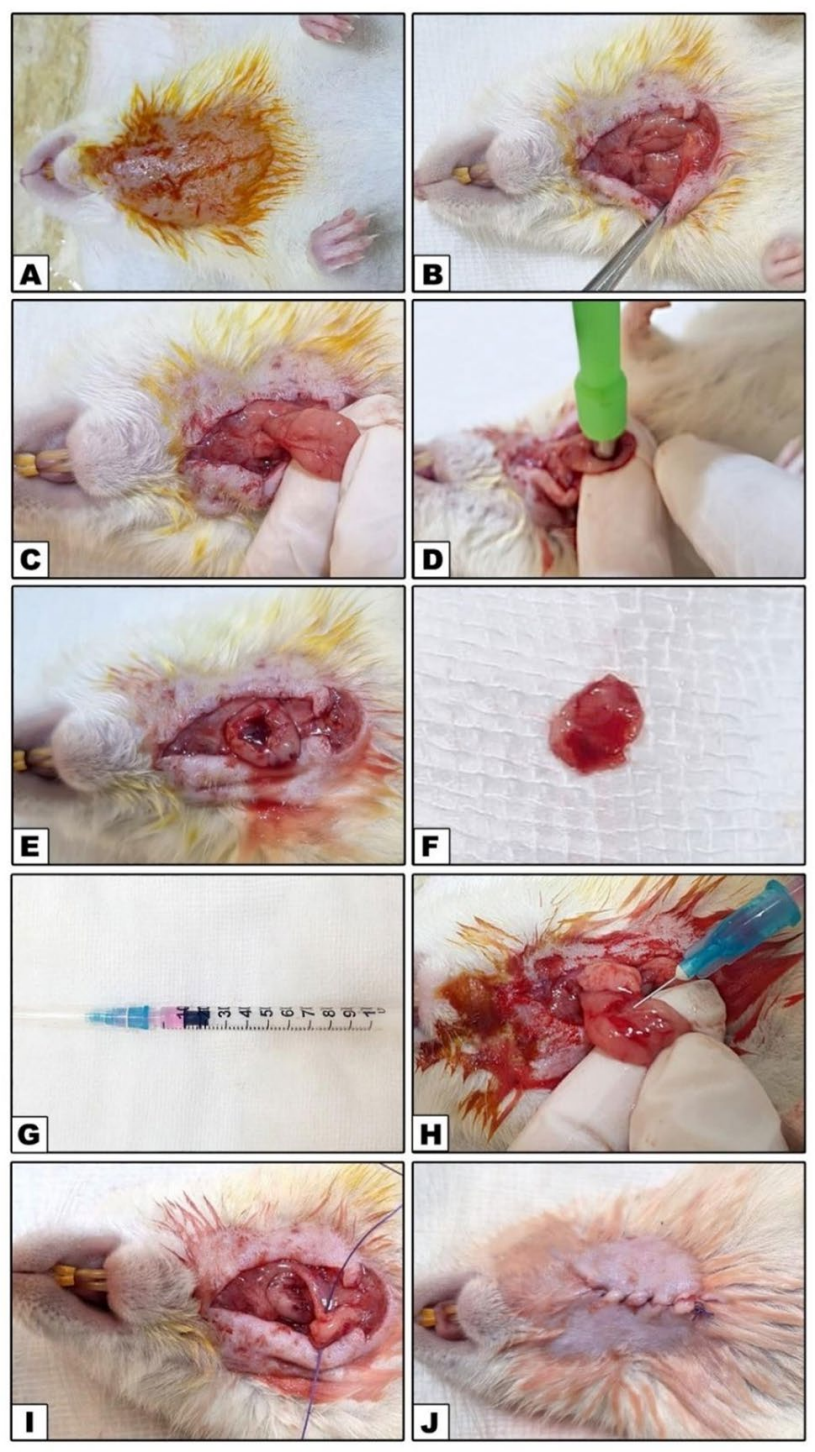
Photographs illustrating surgical procedures showing **A**. shaved skin, **B **incised skin, **C** exposed gland, **D** Defect creation by tissue punch, **E** the prepared defect, **F** the excised tissue, **G** Cells or exosomes suspended in 1 ml PBS, **H** Injection of the gland, **I **&** J**. The two edges of the skin were approximated and sutured

Finally, the skin edges were approached and sutured with 3/0 silk suture installed on a 3/8-inch half-circle needle. Postoperatively, each animal received 0.5 ml of intramuscular Amoxicillin (1 gm/10 ml distilled water, Cat# 7038, Emox, Epico, Cairo, Egypt) as an antibiotic and 0.2 ml of intramuscular Ketoprofen (50 mg, Cat# 471909, Ketophan 50, Sigma-Aldrich, Burlington, US) as an analgesic daily for four consecutive days (Fig. 1).

### Collection of teeth for hDPSCs isolation

Four sounds, impacted third molars were collected from four healthy donors (aged 18–35), for orthodontic purposes, in the Oral Surgery department at the Faculty of Dentistry, Mansoura University, Egypt, after acquiring informed consents in accordance with approval from the Ethical Committee of Mansoura University, Egypt and following the guidelines outlined by the Declaration of Helsinki. The extraction was performed under sever sterilization protocols; the patient rinsed with chlorhexidine mouthwash (Cat# 62135, Epico, Cairo, Egypt). The extracted teeth were thoroughly irrigated for 15 s after extraction with normal saline, ethyl alcohol (70%), and PBS.

After irrigation, the teeth were maintained in sterile falcon tubes containing pasteurized whole milk [21] that was selected as the transport medium because of its physiological pH, osmolarity, and nutrient-rich composition that helped to maintain dental pulp cell viability during the short transport period prior to laboratory processing. In addition, it represented a readily available and cost-effective alternative to specialized culture media for temporary tissue preservation [22].

Pulp harvesting was carried out in the laminar flow hood (Biological safety cabinet class II A2, UNIL@B, Thermo Scientific, Waltham, USA) under meticulous aseptic conditions. All the soft tissues adherent to the teeth surfaces were scraped using curette, then the extracted teeth were irrigated using chlorhexidine followed by PBS. The tooth crown was separated using sterilized dental diamond fissure burs (Mani, Tokyo, Japan) attached to a low-speed hand piece (NSK, USA) under water irrigation to expose the pulp tissue that was gently extirpated utilizing an endodontic H-file # 30 (MANI, Tokyo, Japan). After that, the pulp was rinsed three times with PBS, then minced into small pieces (≤ 0.5 mm) and placed in 10-cm culture dishes [23].

### Isolation and culture of hDPSCs

hDPSCs isolation was carried out following the protocol described by Swanson et al. [24]. The minced dental pulp tissue was enzymatically digested through the application of 3 mg/mL of collagenase type I (Cat # 17018029, Invitrogen, Massachusetts USA) and 4 mg/mL dispase type II (Cat # 17105041, Sigma-Aldrich, Burlington, US) for 45 min at 37 °C.

After digestion, the cell suspension was filtered to remove debris and undigested tissue fragments through a 70 μm cell strainer, followed by centrifugation at 1500 rpm for 5 min using a cooling centrifuge (Sigma-Aldrich, Burlington, US). The resulting pellet was then suspended in 7 ml of Dulbecco’s Modified Eagles Medium low glucose (DMEM; Cat # D5523, Sigma-Aldrich, Burlington, US) supplemented with 10% Fetal bovine serum (FBS; Cat # F9665, Sigma-Aldrich, Burlington, US) and 1% penicillin/streptomycin (Cat# 15070063, GibcoTM, Waltham, USA). The cells were transferred to a tissue culture flask and maintained in an incubator at 37 °C with 5% CO_2_ (Thermo Scientific, Waltham, USA).

The culture medium was refreshed every three days. Cell cultures were monitored each day under an inverted microscope (Olympus, CKX41SF, Tokyo, Japan) until an approximately 80% confluence was reached. The viability and count of cells were evaluated using trypan blue 0.4% (Cat # T10282, Gibco, Waltham, USA). The cells were further sub-cultured at a 1:2 split ratio. Cells were collected for immunophenotypic characterization [25].

### Characterization of hDPSCs

The morphology and adherence of stem cells were evaluated under an inverted microscope. The immunophenotype was determined utilizing flow cytometric analysis.

Third passage stem cells were separated from culture flask using trypsin and harvested, then washed and resuspended in PBS supplemented with 3% FBS containing saturating concentrations (1:100 dilution) of the following anti-human monoclonal antibodies: Fluorescein isothiocyanate CD73-FITC (Cat# B92463, BD Biosciences, California, US), Pacific Blue CD105-PB 450 (Cat# 323219, BioLegend, California, US), Allophycocyanin and Alexa Fluor CD34-APC A750 (Cat# B92463, Beckman Coulter, Krefeld, Germany). The incubation was performed in the dark for 30 min at 4 °C [26]. Unstained cells were utilized as a negative control. A CytoFLEX S Flow Cytometer (Beckman Coulter, California, USA) with CytExpert software was used to assess the expression profiles.

### Isolation of hDPSCs-derived Exos

Upon reaching 70–80% confluency, the culture medium was withdrawn and replaced with α modified minimum essential medium (α-MEM, Cat # 12571063, Sigma-Aldrich, Burlington, US) and cells were incubated for an additional 24 h. The secretome was then collected and subjected to sequential differential centrifugation at 4 °C to remove cells and debris.

Initially, samples were subjected to centrifugation at 300 × g for 10 min to eliminate floating cells, followed by 2,000 × g for 20 min. The supernatant was transferred to ultracentrifuge-compatible polycarbonate tubes and centrifuged at 10,000 × g for 30 min (Ultracentrifuge Optima XPN Ultracentrifuge, Thermo Scientific, Waltham, USA) to remove larger vesicles and apoptotic bodies. Subsequently, the supernatant underwent ultracentrifugation at 100,000 × g for 60 min. The resulting pellet was washed in PBS and centrifuged again at 100,000 × g for 60 min. Finally, the purified exosome pellet was resuspended in PBS and stored at − 80 °C until further analysis [27].

### Exosomes characterization

#### Protein quantification (BCA assay)

Following the manufacturer’s instructions, exosomal protein concentration was evaluated using a bicinchoninic acid (BCA) protein assay kit (Thermo Scientific, Waltham, USA). Bovine serum albumin (BSA) was used to generate a standard curve.

Standard or sample replicate (25 µL) was carefully dispensed into the wells of a microplate. Then, the working reagent (200 µL), which was carefully prepared by combining assay reagents A and B in a 50:1 ratio, was introduced to each well. The plate was incubated at 37 °C for 30 min to promote the development of the colorimetric reaction. Then the absorbance of each well was measured at a wavelength of 562 nm using a plate reader. The concentrations were calculated from the standard curve, and results were displayed in mg/dL [28].

#### Nanoparticle tracking analysis (NTA)

The size distribution and concentration of hDPSCs-Exos were determined using a particle matrix device for Nanoparticle Tracking Analysis (NTA). To ensure that the concentration fell within the optimal detection range for the particle matrix device, typically around 1 × 10⁸ particles/ml, the samples were diluted using sterile PBS, then introduced into the device’s sample chamber utilizing a sterile syringe [29].

Three 60-second videos were recorded for each sample, documenting the Brownian motion of the exosomes in the PBS solution. The Particle Matrix device’s software was employed to analyze these videos, tracking the individual particles’ movement and calculating their size via the Stokes-Einstein equation [29]. Subsequently, exosome purity was determined by dividing the particle concentration obtained from NTA (particles/ml) by the protein concentration measured by BCA assay (mg/ml) [30].

#### Western blot analysis

Exosomal proteins were extracted from 10 µg of exosomes after lysing using Radio-immunoprecipitation Assay buffer (Cat # 89900, RIPA, Thermo Fisher Scientific, Waltham, USA), then mixed with Laemmli sample buffer. The prepared samples were introduced into a 10% sodium dodecyl sulfate polyacrylamide gel (SDS-PAGE; Cat. No. 43423.01, SERVA Electrophoresis GmbH, Heidelberg, Germany). The proteins were transferred from the gel to a polyvinylidene fluoride (PVDF, Cat# IPVH00010, Thermo Scientific, Waltham, USA) membrane utilizing the “transfer sandwich” technique on a BioRad Semi-Dry Blotter following separation.

To detect specific exosomes proteins, the membrane underwent several incubation steps, starting with a 1-hour blocking step in 5% milk in PBS with 0.1% Tween-20 (Cat # 37572, PBS-T, Biosolution, Slivenec, Czech Republic), then incubated with primary antibodies for tetraspanin anti-CD63 (Cat # 556019, BD Biosciences, California, US), and anti-CD81(Cat # SC-23962, Santa Cruz, California, US), and cytosolic anti-syntenin (Cat #GR34015373-9, Abcam, Cambridge, UK) overnight at 4 °C, all diluted to 1:1000 in blocking solution.

After that, the membrane was incubated with goat anti-mouse horseradish peroxidase conjugated secondary antibodies HRP (Cat# 31438, Thermo Scientific, Waltham, USA) at a dilution of 1:10,000 in blocking solution. The membrane was treated for 5 min with ECL™ Prime Western blotting detection reagents to generate chemiluminescence. Finally, the bands were visualized utilizing a BioRad Chemidoc imaging [31].

#### Transmission electron microscopy (TEM)

The obtained cell pellet was resuspended in 50 to 100 µl of 2% paraformaldehyde (PFA, Cat # 158127, Sigma-Aldrich, Burlington, US) for the examination of isolated exosomes.

A 5 µl aliquot of the resuspended pellet was allowed to dry onto Formvar-carbon coated electron microscopy (EM) grids for 20 min. Then, 100 µl drops of PBS were applied to a sheet of Parafilm, and the grids were transferred to these drops using clean forceps for washing.

The grids were subsequently placed in a 50 µl drop of 1% glutaraldehyde (Cat # G6257, Sigma-Aldrich, Burlington, US) for 5 min, then transferred to a 100 µl drop of distilled water for 2 min and finally immersed in a 50 µl drop of uranyl-oxalate solution (pH7) for a duration of 5 min. The grids were then positioned on a 50 µl drop of methyl cellulose-UA for a duration of 10 min at low temperature, then air dried for a duration of 5 to 10 min. The exosomes were visualized with a transmission electron microscope (JEOL JEM-2100) 160 kV at the Electron Microscope Unit, Mansoura University, Egypt [32].

### Administration of hDPSCs and their Exos

Insulin Plastic syringes of 100 IU with 30-gauge (30G) needles were loaded with 1 ml of PBS carrying either 1 × 10^5^ hDPSCs [19] or 20 µg of Exos [20] depending on the treated group. The loaded syringe’s tip was inserted horizontally below the defect edge to inject 0.2 ml of suspension which was repeated five times below the other edges of the defect area.

### Histological evaluation through Hematoxylin and Eosin stain (H&E) [33]

The collected specimens were immediately preserved in 10% neutral buffered formalin for 24 h and subsequently processed for paraffin block production. 4 μm tissue sections were serially sliced with a microtome, subsequently undergoing deparaffinization, rehydration, and staining. H&E was employed to assess histological alterations and regeneration during the healing period. All histological evaluations were performed in a blinded manner, independently, by two experienced examiners unaware of the experimental groups at the time of assessment to minimize observer bias.

### Immunohistochemical (IHC) staining with Anti-Collagen-I antibody [34]

Immunohistochemical staining was performed to evaluate extracellular Collagen-I (COL-I) expression using (Anti-COL-I antibody, Cat # A16891, 1:200, ABcclonal Technology, Woburn, US). Paraffin-embedded Sect.  (5 μm) were deparaffinized, rehydrated by gradient elusion using xylene and ethanol and subjected to antigen retrieval using EDTA. Detection was carried out using the Novolink™ Polymer Detection System, followed by visualization with 3,3′-diaminobenzidine (DAB) (Cat # D12384-5G, Sigma-Aldrich, Burlington, US) as a color developing agent and hematoxylin counterstaining.

Images were analyzed and captured by blinded examiners using a ToupCam^®^ camera mounted on Olympus microscope (model no.: XCAM1080PHA) (×400 magnification) in department of Oral Biology, faculty of dentistry, Mansoura University. Fiji ImageJ (version 2; NIH, Maryland, USA) was then used to analyze six randomly selected fields per slide to determine the percentage area of positive brown staining in relation to the total field area not considering stain intensity. The histological dyes in the microphotographs were digitally separated using the function color deconvolution 2 tool, producing three digital images (a complementary image, H&E, and DAB). Next, the stain-specific area percentage in the DAB and H&E images was determined. Data was described as mean ± standard deviation (SD).

A customized IHC scoring system was developed based on defined cutoff values derived from staining patterns and statistical analysis to ensure objective quantification of COL-I expression [34]. Based on the mean percentage area of staining in the negative control group (considered baseline), COL-I expression was categorized as follows: <1% (minimal–mild), 1–2% (mild–moderate), 2–3% (moderate), 3–4% (moderate–severe), and > 4% (severe).

### Quantitative real time polymerase chain reaction (qRT-PCR) for Aquaporin-5 (Aqua-5) [35]

Submandibular gland tissues were conserved in liquid nitrogen for RNA extraction and quantification of Aqua-5 expression using qRT-PCR. Total RNA was extracted using the acid guanidinium thiocyanate/phenol–chloroform method followed by homogenization in 500 mL TRIzol^®^ Reagent (Cat # 15596018, Invitrogen, California, USA). The NanoDrop™ 2000/2000c Spectrophotometer (Thermo Scientific, USA) was used for verification of RNA purity and concentration.

RNA was reverse transcribed into complementary DNA using a QuantiTect Reverse Transcription Kit (cat # 205311, Qiagen, Germantown, USA) through 5 min of primer annealing at 25 °C, followed by 15 min of reverse transcription at 45 °C, finally 5 min for stopping the reaction at 85 °C. qRT-PCR was then performed for cDNA amplification using HERAPLUS SYBR^®^ Green qPCR (Cat # WF10308001, Willowfort, Birmingham, UK) through initial denaturation for 2 min at 95 °C, that was followed by 40 cycles of 10 s at 95 °C and 30 s at 60 °C.

Expression levels of mRNA target genes: Aqua-5 and housekeeping gene Glyceraldehyde-3 phosphate dehydrogenase (*GAPDH*) were detected. All primers were designed using NCBI Primer-BLAST (https://www.ncbi.nlm.nih.gov/tools/primer-blast/) and purchased from Vivantis Technologies (Selangor Darul Ehsan, Malaysia). The 2 − ΔΔCt method was used to analyse gene expression level [36] then standardized relative to the housekeeping gene *GAPDH*. The primer sequences utilized in this study are listed in (Table 1). All steps were performed using a real-time PCR 7500 fast system (Applied Biosystems; Life Technologies, California, USA).

**Table 1:** Showing primers sequences used in qRT-PCR analysis (Forward (F) and reverse (R) primers)

Primer	Primer sequences
Aquaporin5	F: GGGCCATCTTGTGGGGATT
	R: CCAGTGAGAGGGGCTGAACC
GAPDH	F: TGCCACTCAGAAGACTGTGG
	R: GGATGCAGGGATGATGTTCT

### Statistical analysis

Data analysis was performed using GraphPad Prism 9 (GraphPad Software, San Diego, California, USA). The Shapiro-Wilk test was used to assess the normality of data distribution. Quantitative data were presented as mean ± standard deviation. The Post Hoc Tukey test was used for pairwise comparison after the two-way ANOVA test was used to assess the combined impact of two independent factors on a dependent continuous variable. A probability level of 0.05 was used for statistical significance.

## Results

### hDPSCs characterization results

**Fig. 2:**
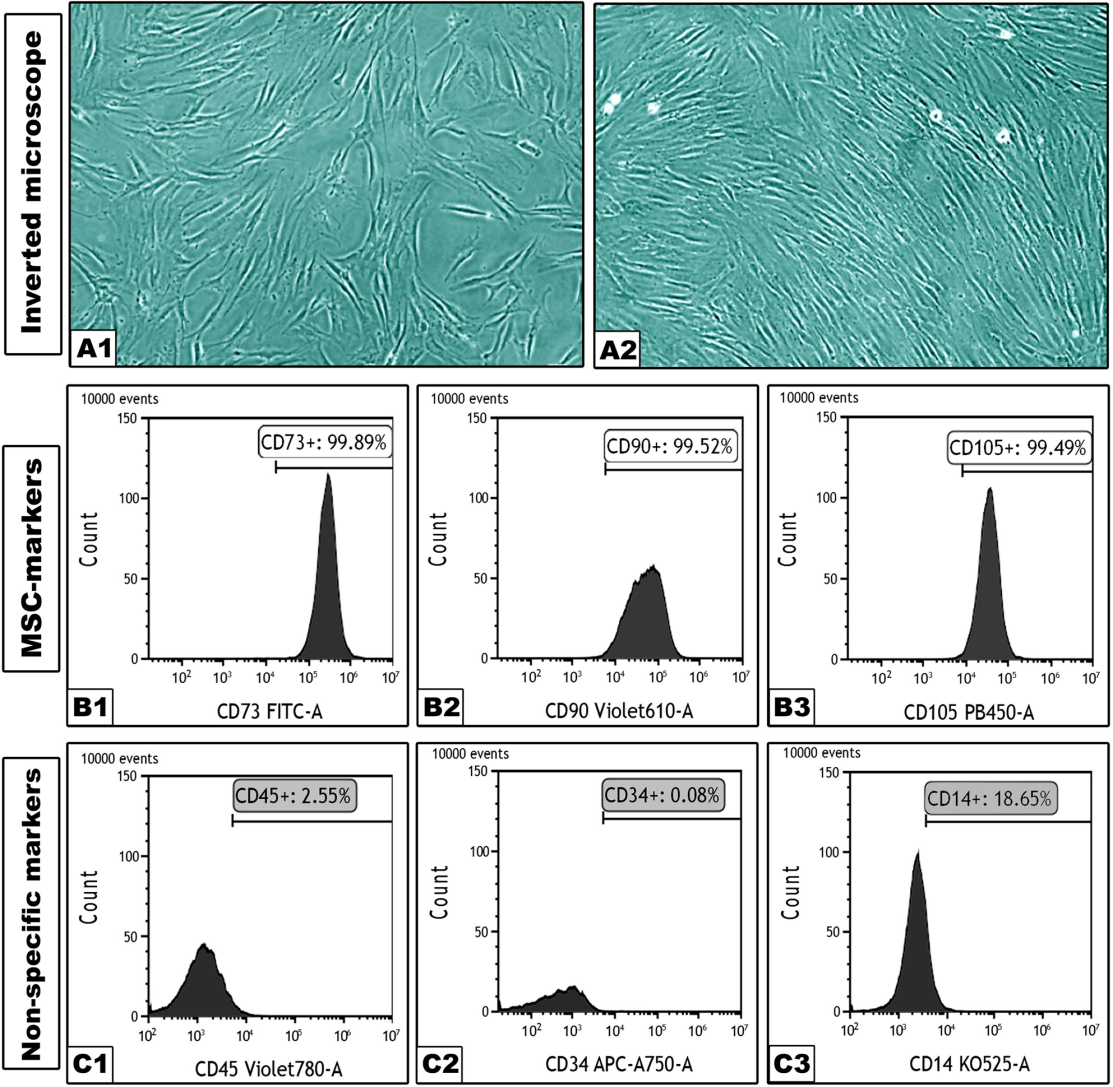
Characterization of hDPSCs Light inverted microscopy images of hDPSCs A1. after 10 days, A2. after 17 days (100×). Flowcytometry histograms for hDPSCs phenotypic characterization utilizing mesenchymal markers B1. CD73, B2. CD90, B3. CD105, and hematopoietic and endothelial lineage markers C1. CD45, C2. CD34, C3. CD14

Under an inverted phase contrast microscope, hDPSCs presented as adherent cells to the plastic walls, displaying a distinctive spindle-shaped and fibroblast-like morphology. Flow cytometric phenotypic surface antigen analysis showed highly positive results for the mesenchymal markers; CD73 (99.89%), CD90 (99.52%), and CD105 (99.49%), while cells expressed negative results for the hematopoietic and endothelial lineage markers CD14 (18.65%), CD34 (0.08%), and CD45 (2.55%) confirming successful isolation of hDPSCs (Fig. 2).

### Characterization of hDPSCs-Exos

The BCA quantification of hDPSCs revealed a protein concentration of 1.006 mg/ml. The NTA showed the original particle concentration to be 3.90 × 10¹¹ particles/ ml and the average particle size (X₅₀) to be 107.6 nm, representing the median particle diameter.

Therefore, the Exos purity was found to be equal 3.88 × 10^8^ mg/particle. This ratio supported the purity of the exosome sample.

Western blot analysis revealed considerable bands for CD63 at the expected molecular weight (~ 53 kDa), CD81 showed distinct bands at (~ 26 kDa), and Syntenin exhibited strong and clear bands at (~ 32 kDa), confirming their presence as a robust Exos marker.

**Fig. 3:**
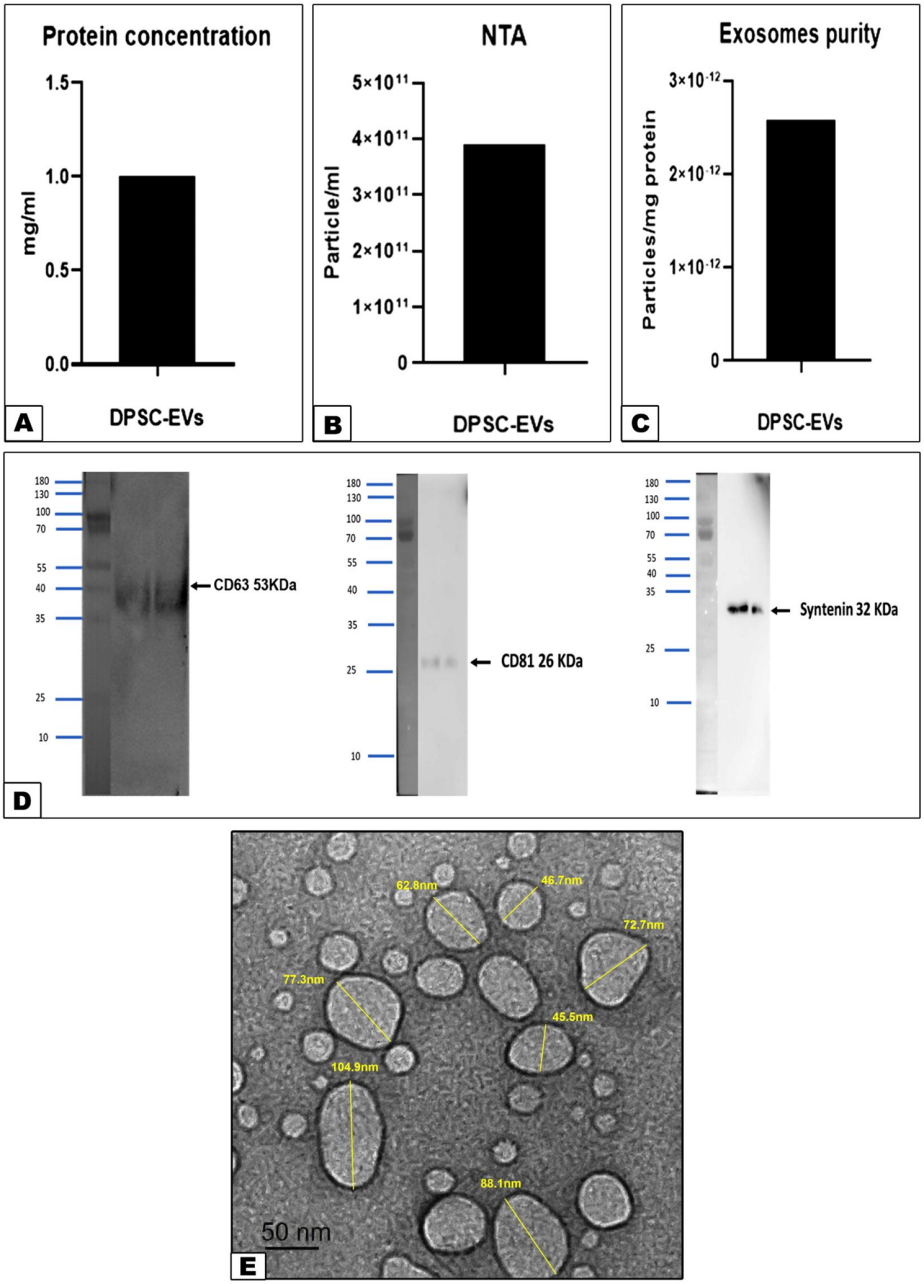
Characterization of hDPSCs-Exos **A.** BCA protein concentration, **B** The particle counts per ml analyzed by NTA, **C** Exosome purity, **D** Western blot analysis results of the exosomal markers (CD63, CD81 and syntenin), **E** Transmission electron microscopy results

TEM examination showed nano-sized Exos manifesting as spherical entities, surrounded by a bilayer lipid membrane. The diameter of hDPCs-Exos ranged from 30 to 150 nm. These results demonstrated the successful isolation of Exos from hDPSCs (Fig. 3, for western blot analysis membranes see [Additional file 1]).

### H&E histological results (Figs. 4 and 5)

**Fig. 4:**
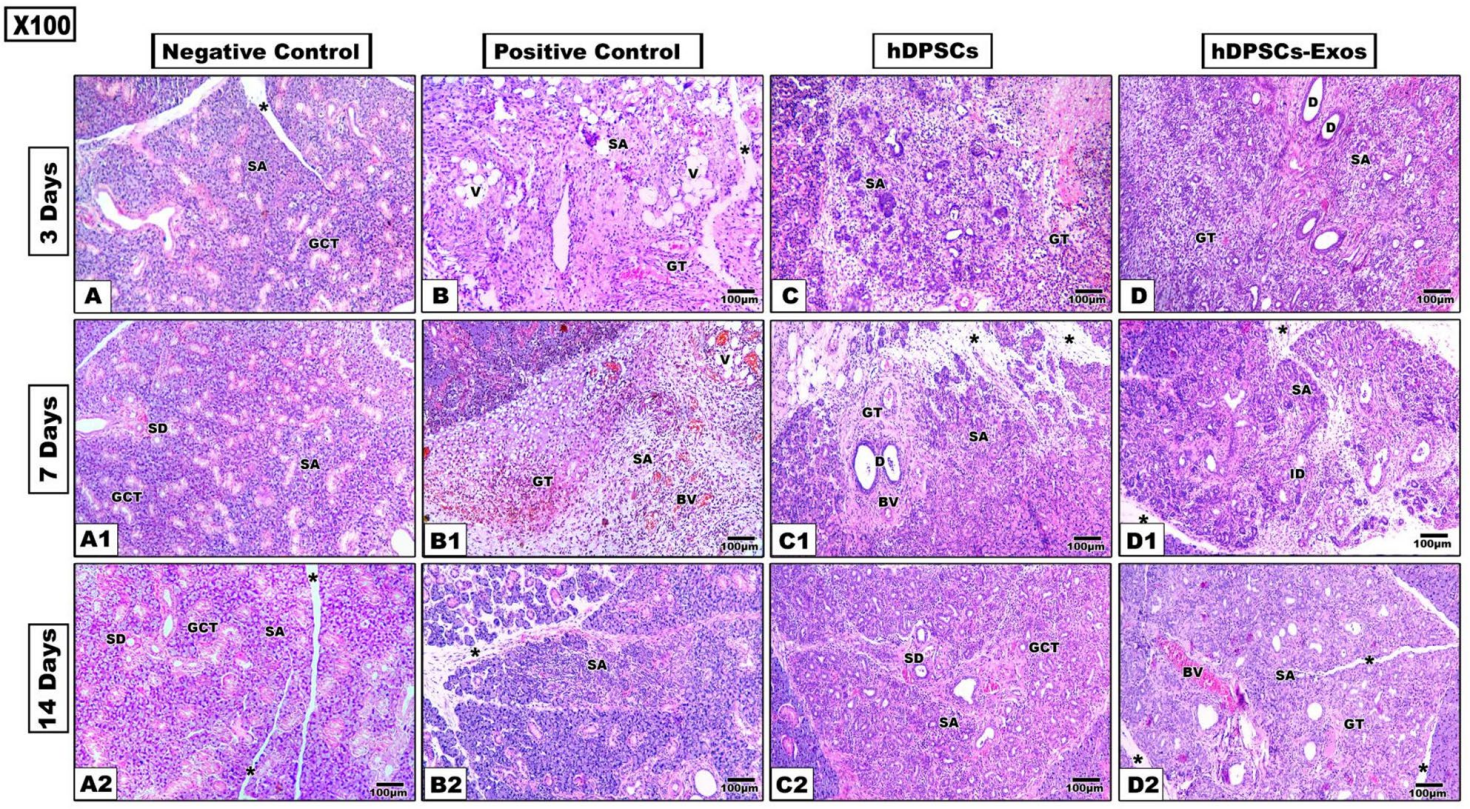
Hematoxylin and Eosin staining results of submandibular salivary glands of different groups (**A**-A2: Negative control, **B**-B2: Positive control, **C**-C2: hDPSCs-treated, **D**-D2: hDPSCs-Exos treated) at 3, 7 and 14 days (magnification 100×). SA: serous acini, SD: striated duct, ID: intercalated duct, D: duct, GCT: granular convoluted tubule, GT: granulation tissue, BV: blood vessel, V: vacuole, Asterix: connective tissue septa

**Fig. 5:**
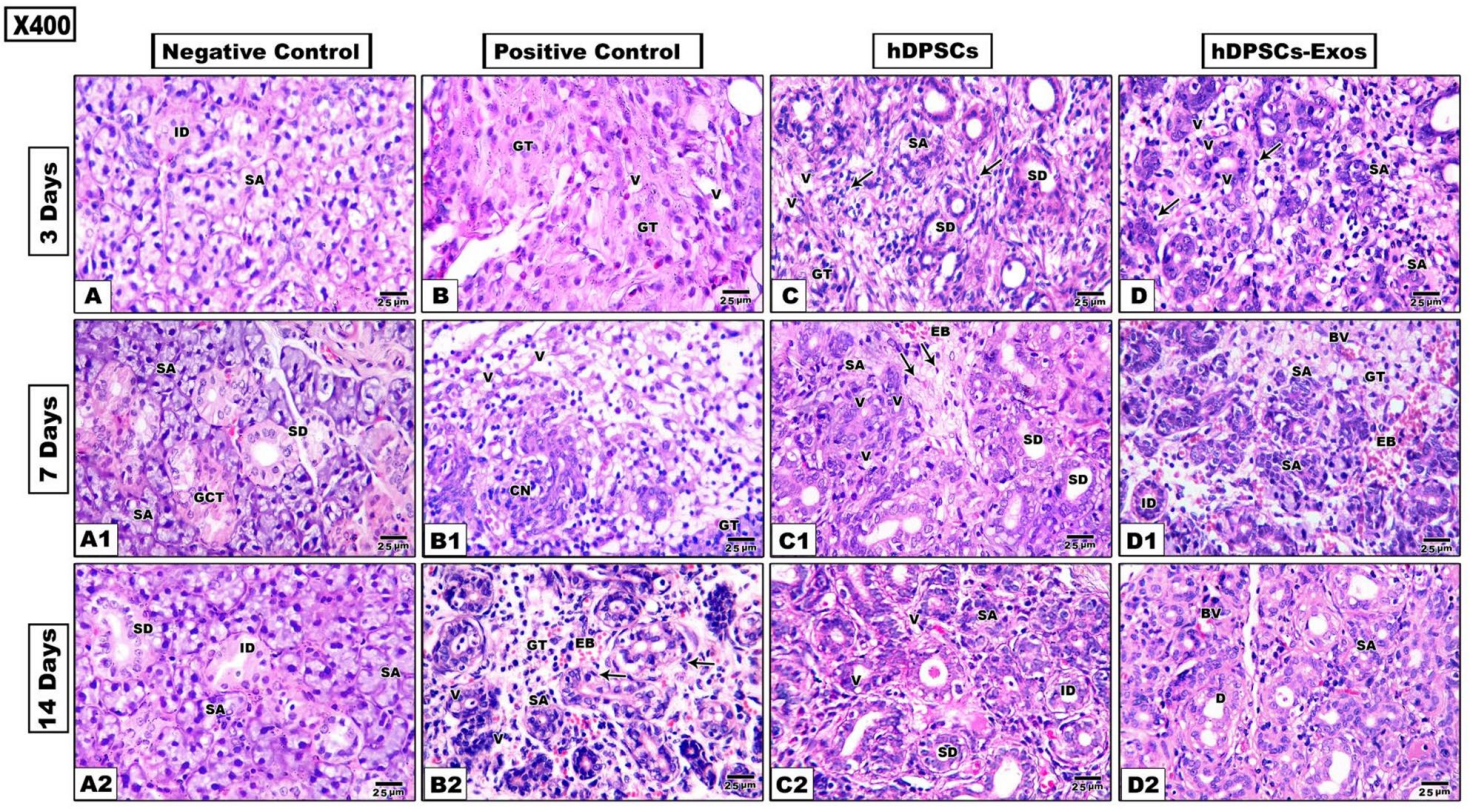
Hematoxylin and Eosin staining results of submandibular salivary glands of different groups (**A**-A2: Negative control, **B**-B2: Positive control, **C**-C2: hDPSCs-treated, **D**-D2: hDPSCs-Exos treated) at 3, 7 and 14 days (magnification 400×). SA: serous acini, SD: striated duct, ID: intercalated duct, D: duct, GCT: granular convoluted tubule, GT: granulation tissue, BV: blood vessel, V: vacuole, EB: extravasated blood, CN: cell nest, Asterix: connective tissue septa

The negative control group (Group I) demonstrated normal glandular architecture throughout the experimental period. The submandibular gland exhibited a well-organized lobular pattern with distinct interlobular and intralobular septa. Serous acini were lined by pyramidal eosinophilic cells with basally located round nuclei bordering narrow lumina, and separated by delicate connective tissue septa. The ductal system, including intercalated ducts, striated ducts, and granular convoluted tubules, displayed normal structural characteristics.

In contrast, the positive control group (Group II) showed marked histopathological alterations. At day 3, the wound area was occupied by fibrous granulation tissue infiltrated with inflammatory cells and containing dilated blood vessels. By day 7, cell nests and few newly formed acini were observed within granulation tissue that was infilterated by inflammatory cells, and accompanied by extravasated blood. The underlying gland exhibited dilated ducts and hemorrhagic areas. At day 14, incomplete regeneration was evident, with sparsely distributed, poorly organized acini embedded in granulation tissue, widened connective tissue septa, cytoplasmic vacuolization, and persistent ductal dilation.

The hDPSCs-treated group (Group III) showed marked histological improvement compared to positive control group. At day 3, granulation tissue with inflammatory infiltration and early acinar and ductal formation was observed. By day 7, increased numbers of newly formed acini and ducts were present, although inflammatory infiltration and ductal dilation persisted. At day 14, clear regenerative features were evident, including aggregated acini with wide lumina and restoration of ductal outlines separated by minimal amounts of granulation tissue, with minor hemorrhagic areas.

The exosome-treated group (Group IV) demonstrated more advanced regeneration. At day 3, granulation tissue contained scattered newly formed acini and dilated ducts with mild inflammatory infiltration. At day 7, the number of regenerated ducts and acini increased. Notably, by day 14, the gland exhibited nearly normal lobular architecture with better developed acini and ducts, absence of granulation tissue, and restoration of structural organization.

### Immunohistochemical staining results with COL-I antibody (anti COL- I)

Positive immunoreactivity for COL-I appeared as brown deposits within the interstitial connective tissue and granulation tissue in all groups. The negative control group showed minimal to mild COL-I expression at all time points. In contrast, the positive control group showed higher COL-I expression compared to all other groups at corresponding time points. The expression was severe at day 3 (4.71%), decreased to moderate–severe at day 7 (3.52%), and further declined to moderate at day 14 (2.47%), indicating partial spontaneous remodeling.

**Fig. 6:**
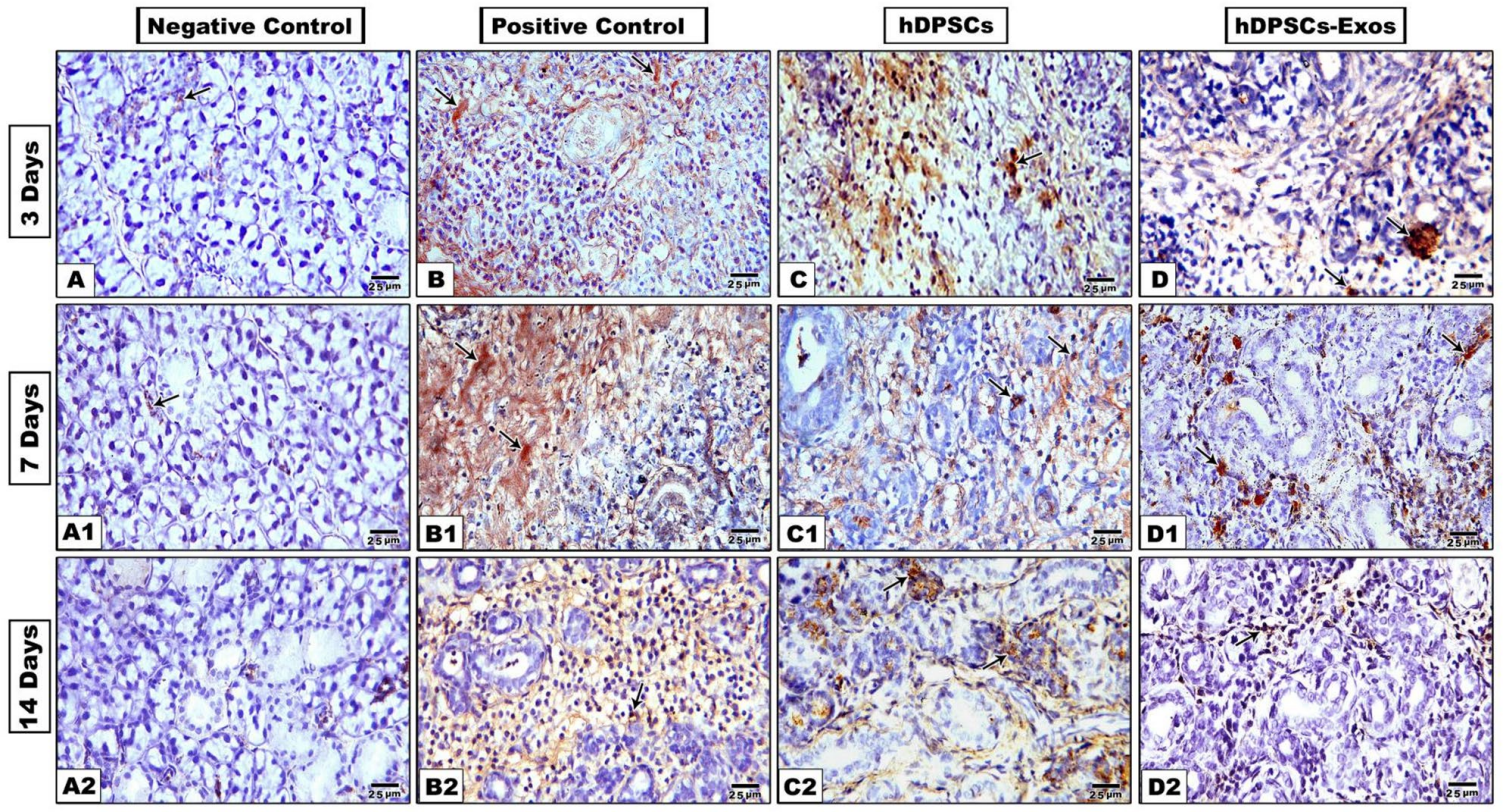
Photomicrograph of submandibular salivary glands immunolabelled with anti-Collagen-I (magnification 400×) of different groups (**A**-A2: Negative control, **B**-B2: Positive control, **C**-C2: hDPSCs-treated, **D**-D2: hDPSCs-Exos treated) at 3, 7 and 14 days (magnification 400×). Arrow: denotes positive brown staining areas

The hDPSCs-treated group demonstrated reduction in COL-I expression over time, decreasing from moderate–severe at day 3 (3.52%) to moderate at day 7 (2.89%) and mild–moderate at day 14 (1.45%). On the other hand, the hDPSCs-Exos treated group exhibited the most pronounced reduction in COL-I deposition as the expression decreased from moderate at day 3 (2.61%) to mild–moderate at day 7 (1.48%) and minimal–mild at day 14 (0.84%) (Fig. 6).

**Fig. 7:**
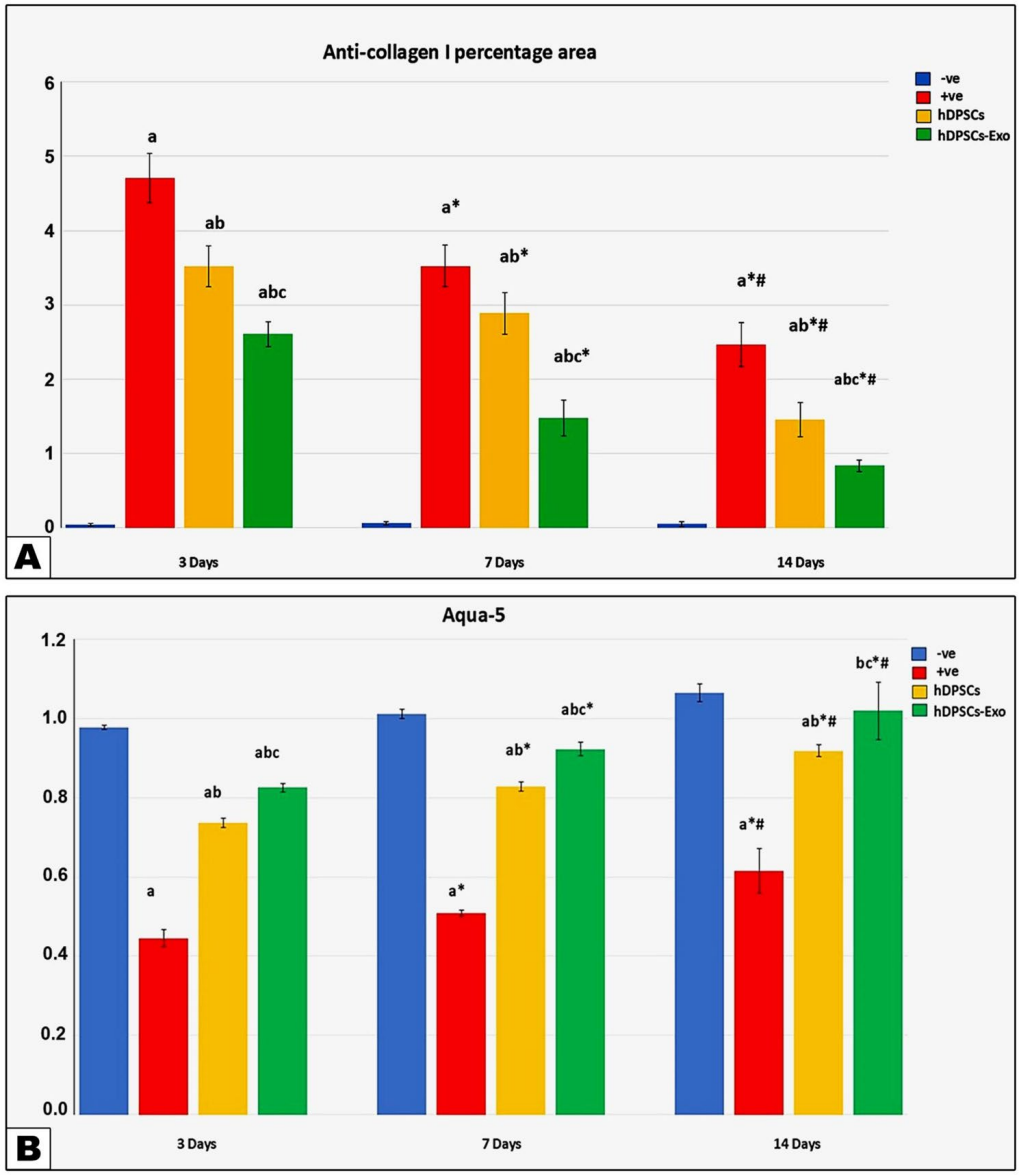
Bar graphs showing the two-way ANOVA statistical analysis followed by post-hoc Tukey’s test for **A**. Anti-Collagen-I immunohistochemical staining results, **B** Aquaporin-5 expression in different groups at different time points. a: Significance Vs negative control within the same time point, b: Significance Vs positive control within the same time point, c: Significance Vs hDPSCs-treated group within the same time point, *: Significance Vs 3 days within the same group, #: Significance Vs 7 days within the same group, at *P* < 0.05

The two-way ANOVA analysis detected a statistically significant interaction between the intervention and time factors [F (6, 60) = 36.5, *P* < 0.0001]. So, analysis of simple main effects for each factor was performed. Considering the intervention factor, the positive control group revealed a significant increase relative to other groups at all time-points. The hDPSCs-treated group revealed significant decrease in relation to positive control group while it was still significantly higher than both negative control and exosome-treated groups at all time-points. The hDPSCs-Exos treated group exhibited significantly lower values relative to other experimental groups, but still significantly higher than negative control group indicating the most pronounced restoration of glandular architecture. Regarding the time factor, there was a statistically significant decrease in the positive mean percentage area within each group through the three time points. On the other hand, the negative control group showed a non-significant difference (Fig. 7A; Table 2).

**Table 2:** Post hoc Tukey test for pairwise comparison of factors affecting the Anti-collagen I positive mean percentage area.

Time	Negative control	Positive control	hDPSCs	hDPSCs – Exo
3 days	**0.04 ± 0.02**	**4.71 ± 0.33**	**3.52 ± 0.27**	**2.61 ± 0.17**
Post hoc		*P* -VE < 0.0001*	*P* -VE < 0.0001*	*P *-VE < 0.0001*
			*P *+ VE < 0.0001*	*P *+ VE < 0.0001*
				*P* Cell < 0.0001*
7 days	**0.06 ± 0.02**	**3.52 ± 0.28**	**2.89 ± 0.28**	**1.48 ± 0.24**
Post hoc	*P*3 > 0.9999	*P*3 < 0.0001*	*P*3 = 0.0003*	*P*3 < 0.0001*
		*P -*VE < 0.0001*	*P *-VE < 0.0001*	*P* -VE < 0.0001*
			P + VE = 0.0003*	*P* + VE < 0.0001*
				*P* Cell < 0.0001*
14 days	**0.05 ± 0.03**	**2.47 ± 0.30**	**1.45 ± 0.23**	**0.84 ± 0.08**
Post hoc	*P*3 > 0.9999	*P*3 < 0.0001*	*P*3 < 0.0001*	*P*3 < 0.0001*
	*P*7 > 0.9999	*P*7 < 0.0001*	*P*7 < 0.0001*	*P*7 = 0.0002*
		*P *-VE < 0.0001*	*P *-VE < 0.0001*	*P *-VE < 0.0001*
			*P *+ VE < 0.0001*	*P *+ VE < 0.0001*
				*P* Cell = 0.0005*

Data was presented as Mean±SD, *P*: Probability, *: significance <0.05, *P*3: significance Vs 3 days, *P*7: significance Vs 7 days, *P* -VE: significance Vs Negative control, *P* +VE: significance Vs Positive control, *P* Cell: significance Vs hDPSCs-treated group

### qRT-PCR analysis results of Aquaporin-5 gene expression

The two-way ANOVA analysis for qRT-PCR of Aqua-5 gene expression revealed a statistically non-significant interaction between the intervention and time factors [F (6, 24) = 2.303, *P* = 0.0674]. So, the main effect analysis for each factor was evaluated separately. Although the interaction between intervention and time did not reach statistical significance, day-by-day specific Tukey post-hoc comparisons were retained for descriptive purposes, to facilitate clinical interpretation of group differences at each time point and to maintain visual and clinical consistency with the obtained COL-I data.

Concerning the intervention factor, a statistically significant main effect was observed. [F (3, 24) = 482.9, *P* < 0.0001], where the positive control group showed significantly lower expression compared to all the other groups. On the other hand, the hDPSCs-treated group revealed a significant increase in relation to the positive control group but remained significantly lower than the negative control and hDPSCs-Exos treated groups at all time points. The hDPSCs-Exos treated group revealed a significant increase relative to positive control and hDPSCs-treated group. Relative to the negative control group, there were significant decreases 3 and 7 days after treatment, while after 14 days the difference became non-significant.

Concerning the time factor, a significant main effect was observed [F (2, 24) = 2.03, *P* < 0.0001]. All the experimental groups showed significant increase over the time. On the other hand, the negative control group showed non-significant differences between the three timepoints (Fig. 7B; Table 3).

**Table 3:** Post hoc Tukey test for pairwise comparison of factors affecting the Aqua-5 gene expression level

Time	Negative Control	Positive Control	hDPSCs	hDPSCs – Exo
3 days	0.98 ± 0.01	**0.45 ± 0.02**	**0.74 ± 0.01**	**0.83 ± 0.01**
Post Hoc		*P* -VE < 0.0001*	*P *-VE < 0.0001*	*P* -VE < 0.0001*
			*P* + VE < 0.0001*	*P *+ VE < 0.0001*
				*P* Cell = 0.044*
7 days	**1.01 ± 0.01**	**0.51 ± 0.01**	**0.83 ± 0.01**	**0.92 ± 0.02**
Post Hoc	*P*3 = 0.9462	*P*3 = 0.0236*	*P*3 = 0.0318*	*P*3 = 0.0193*
		*P* -VE < 0.0001*	*P *-VE = 0.0417*	*P* -VE = 0.0417*
			*P* + VE < 0.0001*	*P *+ VE < 0.0001*
				*P* Cell = 0.027*
14 days	**1.06 ± 0.02**	**0.61 ± 0.06**	**0.92 ± 0.02**	**1.02 ± 0.07**
Post Hoc	*P*3 = 0.0504	*P*3 < 0.0001*	*P*3 < 0.0001*	*P*3 < 0.0001*
	*P*7 = 0.5799	*P*7 = 0.0189*	*P*7 = 0.0417*	*P*7 = 0.0205*
		*P *-VE < 0.0001*	*P *-VE = 0.0002*	*P* -VE = 0.7718
			*P* + VE < 0.0001*	*P* + VE < 0.0001*
				*P* Cell = 0.0131*

Data was presented as Mean±SD, *P*: Probability, *: significance <0.05, *P*3: significance Vs 3 days, *P *7: significance Vs 7 days, *P* -VE: significance Vs Negative control, *P* +VE: significance Vs Positive control, *P *Cell: significance Vs hDPSCs-treated group

## Discussion

SGs are frequently affected by benign and malignant tumors that are commonly managed by surgical excision, often resulting in hyposalivation and compromised quality of life. Tissue engineering has emerged as a promising regenerative strategy to restore glandular structure and function [37]. So, the present study aimed to compare between the regenerative effect of the hDPSCs and their exosomes on surgically induced defects of submandibular salivary gland.

Adult male albino rats were used as an experimental model due to the structural and functional similarity of their submandibular glands to humans and their reliability in research [38]. A standardized partial surgical defect was created to simulate localized tissue loss following tumor excision, allowing assessment of regenerative responses within the remaining glandular tissue rather than complete organ destruction. This model is particularly suitable for evaluating wound healing, acinar regeneration, and ductal reorganization [19].

The use of xenogeneic hDPSCs and their exosomes in rat model was based on the fact that DPSCs exhibit negative immune regulation, low immunogenicity, and immune tolerance. hDPSCs express low levels of MHC-II and co-stimulatory molecules, thereby reducing the risk of xenogeneic immune rejection as supported by several studies [39–41]. Moreover, hDPSCs have been used in several studies on immunocompetent rats and reported positive results without immunorejection [42, 43]. In addition, hDPSC-derived exosomes are largely non-immunogenic cell-free vesicles that exert their therapeutic effects through paracrine signaling and immune modulation [44], which may explain the absence of evident rejection-related responses observed in the present study.

The selected doses of hDPSCs and their Exos were based on previous preclinical studies demonstrating effective SGs regeneration without adverse effects [19, 20]. Intraglandular injection was adopted to ensure localized delivery of therapeutic agents directly into the defect site, maximizing local bioavailability while minimizing systemic exposure. It also requires a much smaller dose of medication [45].

Histologically, the negative control group maintained normal glandular architecture, which was in accordance with Amano et al. [16] and Sakr M [46]. The presence of intact acini and ducts in this group validated the experimental model, as it provided a baseline for comparing injury-induced alterations in subsequent groups. It also confirmed that the histological preparation and staining techniques were appropriate for assessing the structural changes induced by injury and treatment.

The positive control group showed persistent granulation tissue, inflammatory infiltration, dilated ducts, acinar disorganization, and cytoplasmic vacuolization, indicating limited spontaneous regenerative capacity and a tendency toward fibrotic healing. These findings suggest that endogenous repair mechanisms in salivary glands are insufficient for complete acinar restoration, which was consistent with Kobayashi et al. [47] and Zuo et al. [48] who reported minimal acinar cell proliferation, fibrosis, ductal hyperplasia, and partial compensation by a limited population of ductal progenitor cells.

The widespread formation of granulation tissue and dilated ductal structures observed in this study were also in agreement with the findings of Abd El-Latif et al. [19] who demonstrated that the defect cavity was filled with a heavy condensation of granulation tissue with only partial regeneration of few serous acini. The cytoplasmic vacuolization observed in acinar and ductal cells could be explained according to Shubin et al. [49] who referred it to the reduction in the water content in the cytoplasm of apoptotic cells.

Subsequently, the cells compensate for this by swelling, vacuolization and formation of lipid-filled or autophagic vacuoles that may progress to death of cell.

The hDPSCs-treated group demonstrated marked structural improvement over time, characterized by increased acinar and ductal formation and partial restoration of glandular organization. These effects could be attributed to the paracrine signaling effect as hDPSCs secrete multiple growth factors such as vascular endothelial growth factor (VEGF), fibroblast growth factor-2 (FGF-2), platelet derived growth factor (PDGF), insulin like growth factor-1 (IGF-1), and transforming growth factor beta (TGF-β) that promote angiogenesis, proliferation, and extracellular matrix remodeling [50].

These outcomes were in accordance with Al-Serwi et al. [43] and Li et al. [51] who demonstrated that stem/progenitor cell transplantation into defected SGs increased proliferative activity and contributed to structural and functional recovery. Also, Najafi et al. [52] reported that the local injection of MSCs in a necrotic submandibular gland model, increased secretory activity of both serous and mucous cells, increased proliferation, and improved histologic structure within 2 weeks post-transplantation confirming the regenerative and reparative effect of stem cells.

However, the incomplete structural organization and residual vacuolization within this group could be attributed to the fact that cell survival and engraftment could compromised within the hostile microenvironment of injured tissue, where oxidative stress, hypoxia, and inflammation can limit transplanted cell efficacy as Hu et al. [53] reported that the transplanted cells are exposed to high levels of reactive oxygen species (ROS) in addition to hypoxia and nutritional deprivation in damaged tissue. Moreover, Buja LM and Vela D [54] reported that the inflammation within the injured tissue tend to recruit immune cells such as macrophages and neutrophils, resulting in phagocytosis and destruction of the transplanted stem cells.

Notably, the hDPSCs-Exos treated group demonstrated superior regenerative outcomes. By day 14, glandular architecture was nearly restored, with organized acini, well-defined ducts, and absence of granulation tissue. In consistent with the present study, Zakaria et al. [55] and Desouky et al. [56] reported that the bone marrow stem cells (BMSCs) derived exosomes treated group showed enhanced cell proliferation, reduced oxidative stresses, cell apoptosis and marked tissue restoration, with more uniform duct systems and acini.

The reduced presence of vacuolated cytoplasm and pyknotic nuclei in group IV suggests that Exos also contributed to cytoprotection and tissue repair which agreed with AbuBakr et al. [20] who evaluated the effect of BMSCs-Exos on submandibular SGs of diabetic rats and demonstrated that exosome treatment reduced acinar degeneration, vacuolization and apoptotic changes and restored normal tissue structure.

The enhanced therapeutic effect of the Exos may be attributed to their ability to deliver bioactive proteins, lipids, mRNAs, and microRNAs that modulate angiogenesis, suppress apoptosis, regulate inflammation, and promote tissue remodeling [57]. Unlike cell transplantation, Exos bypass challenges related to poor cell survival, immune-mediated destruction, and engraftment failure, supporting the concept of cell-free regenerative therapy as a safer and logistically easier alternative.

Regarding ECM remodeling, COL-I expression served as an indicator of tissue remodeling as the pattern of COL-1 expression reflects the balance between fibrosis and regeneration as persistent or excessive collagen I accumulation is often associated with fibrotic healing, whereas organized and balanced deposition indicates regenerative remodeling [58].

The immunohistochemical staining results for COL-I in the negative control group revealed minimal or faint immunoreactivity, limited to normal interstitial connective tissue, indicating normal glandular architecture without fibrosis which was in agreement with Tumer et al. [14].

On the other hand, the positive control group showed persistently elevated COL-I deposition that was significantly higher than other treated groups at all time periods which could be attributed to the sustained inflammation and TGF-β–mediated fibroblast activation as TGF-β promotes the production of collagen and fibronectin, two essential proteins in the progression of fibrosis which came in agreement with Ibrahem et al. [59], Woods et al. [60] and Altrieth et al. [61].

In treated groups, COL-I expression progressively declined over time, reflecting a shift from fibrotic repair toward regenerative remodeling which was in agreement with McArthur et al. [62] and Wang et al. [63], who reported that COL-I expression peaks in the early phase of injury reflecting early fibroblast activity and ECM deposition and subsequently declines indicating resolution of inflammation and maturation of the repaired tissue.

In the hDPSCs-treated group, COL-I expression significantly decreased over time, indicating antifibrotic and pro-regenerative effects likely mediated through paracrine secretion of immunomodulatory cytokines that regulate extracellular matrix remodeling which was also consistent with Abd El-Latif et al. [19].

The exosome-treated group demonstrated the most pronounced antifibrotic effect, with COL-I levels approaching baseline by day 14. This finding aligned with AbuBakr et al. [20] who demonstrated significant suppression of COL-I in SG injury following BMSCs- Exos treatment. This highlights the potent bioactivity of Exos as key mediators of tissue repair and remodeling as MSC-derived Exos have the ability to modulate fibroblast, keratinocytes, and endothelial cells activity and balance collagen synthesis and degradation.

Zhou et al. [64] reported that adipose stem cells derived Exos (ADSCs-Exos) were recruited to wound sites to induce COL-I/III formation in the early healing period and prevented collagen deposition in the late period, thereby supporting scar-limited healing.

These results support the notion that cell-free exosome therapy provides a more controlled and sustained regenerative effect compared to direct stem cell transplantation.

Aqua-5 was selected as a molecular biomarker for potential functional recovery due to its critical role as a water channel protein essential for saliva secretion and acinar cell activity. qRT-PCR analysis of Aqua-5 gene expression provided molecular evidence supporting the observed structural regeneration. The negative control group maintained stable physiological baseline expression, whereas the positive control group showed significantly reduced Aqua-5 levels, indicating acinar degeneration and impaired glandular function. These findings were consistent with Taher et al. [65] demonstrating diminished Aqua-5 expression in untreated injured glands.

Both treated groups exhibited significantly higher Aqua-5 expression than the untreated control. In the hDPSCs-treated group, Aqua-5 expression increased progressively over time, suggesting regenerative potentially. However, the recovery remained slower compared to the exosome-treated group, possibly due to limited survival and integration of transplanted cells within the injured microenvironment.

Conversely, the exosome group revealed the greatest upregulation by day 14, reaching levels comparable to the negative control. These findings aligned with Hu et al. [66] and Guo et al. [67] demonstrating that intravenous injection of DPSCs-Exos and ADSCs- Exos enhanced SGs function, and increased Aqua-5 expression, and reduced glandular inflammation. However, the superior results detected in the exosomes group may be in part be due to the contribution of adjacent healthy tissue in addition to the regeneration occurring at the injury site.

The findings of the present study further support the clinical translational potential of hDPSCs-exosomes as a promising cell-free regenerative therapy. Unlike stem cell transplantation, exosome-based therapy avoids several major challenges associated with living-cell administration, including poor cell survival, potential vascular embolization, uncontrolled differentiation, and ectopic tissue formation. Moreover, exosomes exhibit lower immunogenicity, greater storage stability, and easier large-scale production and quality control [68]. These advantages, combined with their ability to recapitulate many of the paracrine regenerative and immunomodulatory effects of their parent stem cells, position hDPSC-derived exosomes as an attractive and potentially safer alternative for future clinical applications in salivary gland regeneration.

Among the limitations of the present study is the limited follow-up period for only two weeks which could be extended in future studies to assess the long-term effect and detect any possible side effects that might surface. Moreover, the limited sample size may reduce the statistical power and generalizability of the results. The detected CD14 expression of 18.65% positivity is relatively high and may reflect minor co-isolation of non-mesenchymal cells. This may be attributed to the heterogeneous cellular composition of primary dental pulp cultures and the early passage at which characterization was performed.

Eventhough the use of hDPSCs in immunocompetent rats has been supported by previous research, their use without immunosuppression, cell tracking or monitoring of the immune response may have affected the results of stem cell treated group, therefore this point should be addressed in more details before clinical translation of the obtained results. Additionally, only single dose of either hDPSCs or their Exos was tested which was based on previous studies, however upcoming studies comparing different doses could be addressed.

## Conclusions

Both hDPSCs and their exosomes had a significant positive impact on submandibular salivary gland regeneration after injury with significant decrease in collagen I expression reflecting the shift toward tissue remodeling and regeneration rather than fibrosis. The tested therapies managed to restore not only the structure, but also the molecular biomarkers for functioning of the regenerated tissue that was confirmed by the significant increase in aquaporin-5 levels. The exosomes treated group showed superior structural and molecular outcomes supporting the emerging role of exosome-based therapies in overcoming the limitations of direct cell transplantation, while providing a safer, more reliable, and clinically translatable approach. 

## Supplementary Information

Supplementary Material 1.

## Data Availability

All data generated or analyzed during this study are included in this published article.

## Abbreviations

ADSCs-Exos: Adipose stem cells derived Exos
ANOVA: Analysis of variance
Aqua-5: Aquaporin-5
ARRIVE: Animal Research: Reporting of In Vivo Experiments
BCA: Bicinchoninic acid
BMSCs -Exos: Bone marrow stem cells derived exosomes
BSA: Bovine serum albumin
cDNA: Complementary DNA
COL-I: Collagen-I
DAB: Diaminobenzidine
DMEM: Dulbecco’s Modified Eagles Medium low glucose
ECM: Extra cellular matrix
EDTA: Ethylene diamine tetra acetic acid
FBS: Fetal bovine serum
GCT: Granular convoluted tubules
H&E: Hematoxylin and Eosin stain
hDPSCs: Human dental pulp stem cells
hDPSCs-Exos: Human dental pulp stem cells derived exosomes
IHC: Immunohistochemical analysis
MEM: Modified minimum essential medium
MERC: Medical Experimental Research Center
ml: Milliliter
mm: Millimeter
MSCs: Mesenchymal stem cells
MU-ACUC: Mansoura University Animal Care and Use Committee
µg: Microgram
µm: Micrometer
NTA: Nanoparticle tracking analysis
PBS: Phosphate buffered saline
PVDF: Polyvinylidene fluoride
qRT-PCR: Quantitative real time polymerase chain reaction
RIPA: Radioimmunoprecipitation assay
SDS-PAGE: Sodium dodecyl sulfate polyacrylamide gel
SG: Salivary gland
TEM: Transmission electron microscopy
