# Supplementary material for: Regenerative capacity of human dental pulp stem cells versus their exosomes on surgically induced submandibular gland defects in rats
Source: BMC Oral Health. 2026 Jul 22;26:1332. doi: 10.1186/s12903-026-09337-9 (PMC13397795; doi:10.1186/s12903-026-09337-9)
Supplement: Supplementary file 1 — Supplementary Material 1. [file 12903_2026_9337_MOESM1_ESM.pdf]

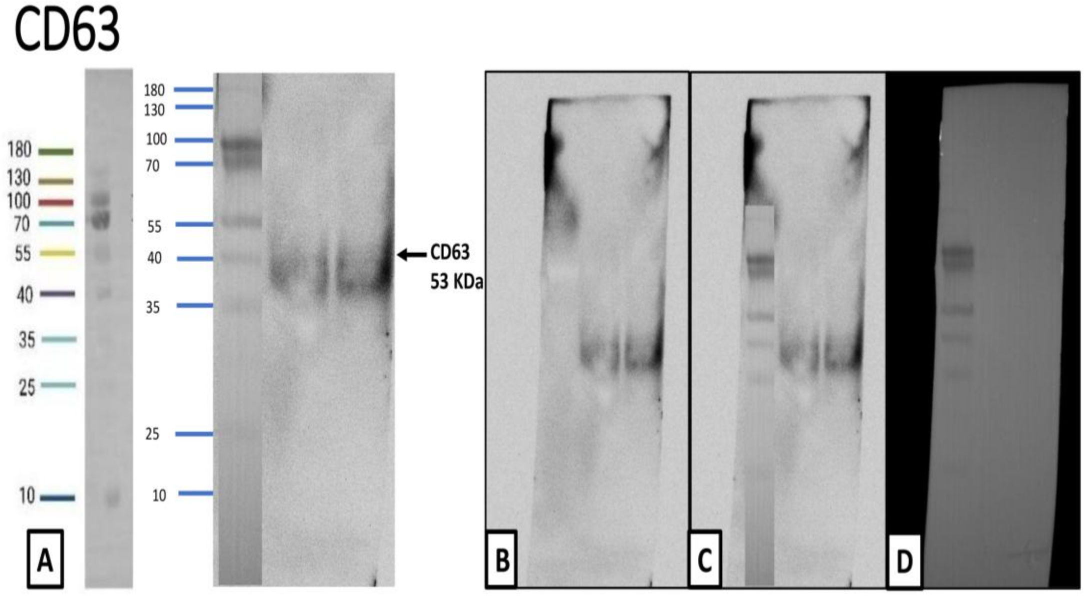

Figure (1): Photomicrograph showing **western blot analysis results of the exosomal markers (CD63) in hDPSC-derived Exos (A)** CD63 hDPSCs-Exos with ladder, (B) CD63 without ladder, (C) CD63 with ladder, (D) Protein LADDER for CD63.

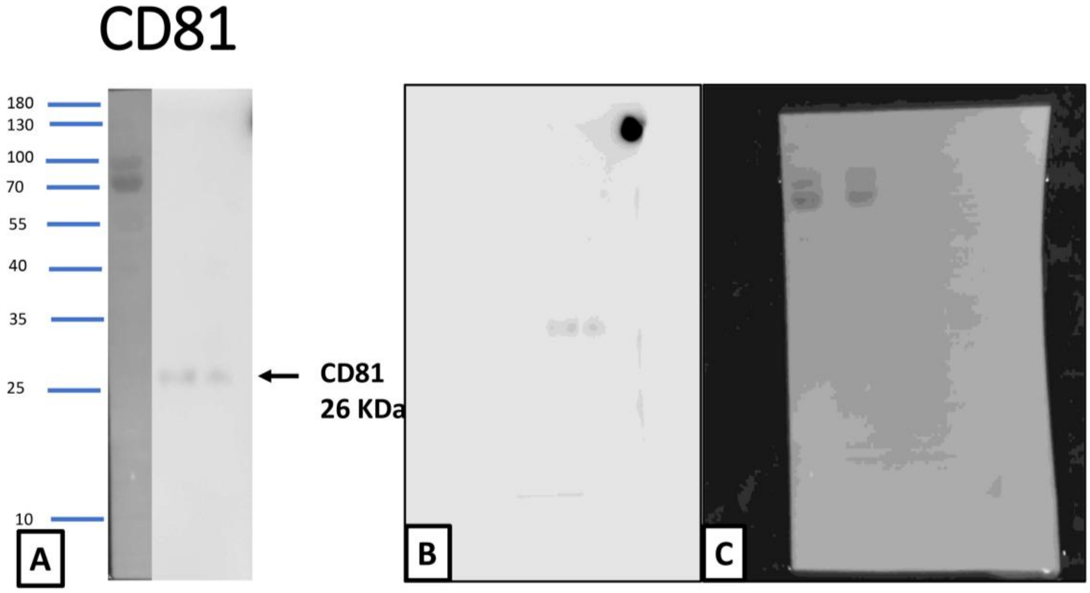

Figure (2): Photomicrograph showing **western blot analysis results of the exosomal markers (CD81) in hDPSC-derived Exos (A)** CD81 hDPSCs-Exos with Ladder, (B) CD81 without ladder, (C) Protein LADDER for CD81.

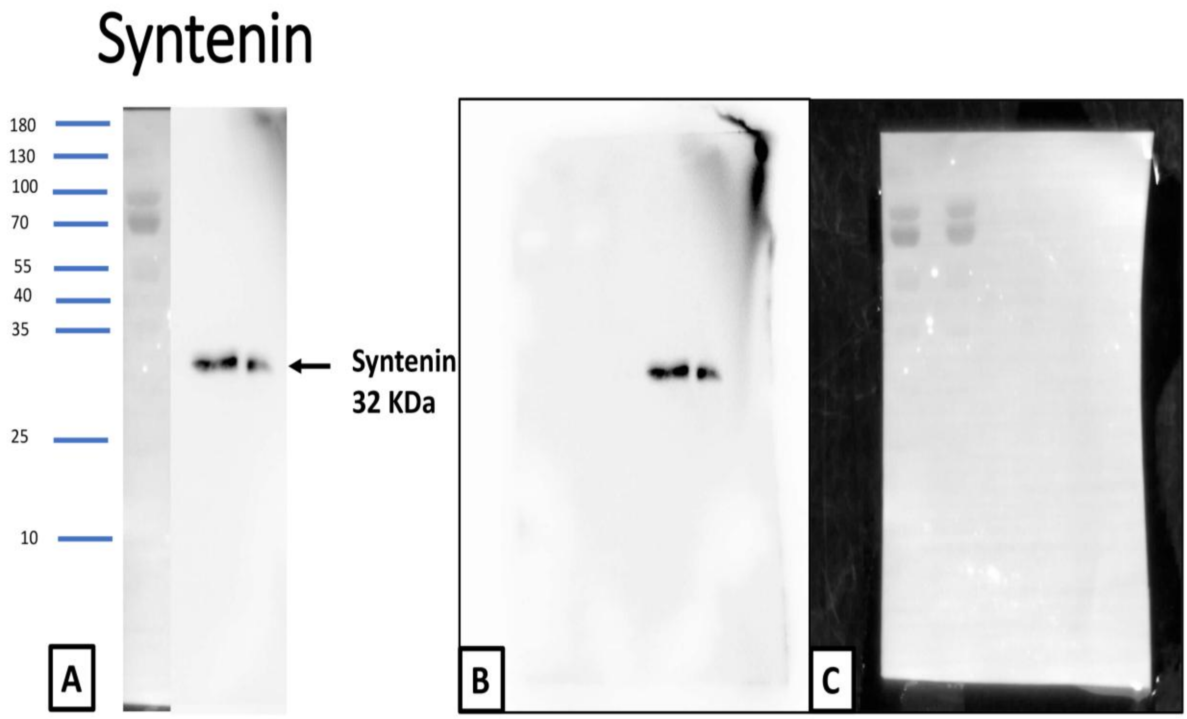

Figure (3): Photomicrograph showing **western blot analysis results of the exosomal markers (Syntenin) in hDPSC-derived Exos** (A) Syntenin hDPSCs-Exos with ladder, (B) Syntenin without ladder, (C) Protein LADDER for Syntenin
